# Differential myofiber-type transduction preference of adeno-associated virus serotypes 6 and 9

**DOI:** 10.1186/s13395-015-0064-4

**Published:** 2015-11-10

**Authors:** Muhammad Riaz, Yotam Raz, Elizabeth B. Moloney, Maaike van Putten, Yvonne D. Krom, Silvere M. van der Maarel, Joost Verhaagen, Vered Raz

**Affiliations:** Department of Human Genetics, Leiden University Medical Center, Building 2, Room R3-17, Einthovenweg 20, 2333 ZC Leiden, The Netherlands; Department of Regeneration of Sensorimotor Systems, Netherlands Institute for Neuroscience, Royal Dutch Academy of Sciences, Amsterdam, The Netherlands; Department of Molecular and Cellular Neurobiology, Center for Neurogenomics and Cognition Research, Vrije Universiteit Amsterdam, Amsterdam, The Netherlands

**Keywords:** Skeletal muscle, Adeno-associated viral vectors, AAV serotypes, Gene therapy, Myofiber types

## Abstract

**Background:**

Gene therapy strategies are promising therapeutic options for monogenic muscular dystrophies, with several currently underways. The adeno-associated viral (AAV) vector is among the most effective gene delivery systems. However, transduction efficiency in skeletal muscles varies between AAV serotypes, with the underlying factors poorly understood. We hypothesized that myofiber-specific tropism differs between AAV serotypes.

**Methods:**

We developed a quantitative histology procedure and generated myofiber pattern maps for four myosin heavy chain (MyHC) isotypes. We compared myofiber pattern maps between AAV6 or AAV9 injected tibialis anterior muscle in mice. We correlated MyHC expression with AAV-derived green fluorescence protein (GFP) expression using statistical models.

**Results:**

We found that MyHC-2x expressing myofibers display a significantly higher preference for AAV transduction, whereas MyHC-2b expressing myofibers negatively correlated with AAV transduction. In addition, we show that AAV9-mediated transduction is enriched in myofibers expressing MyHC-1 and MyHC-1/2a. Moreover, AAV9-mediated transduction can predominantly be predicted by the expression of MyHC isotypes. In contrast, AAV6 transduction can be predicted by myofiber size but not by myofiber types.

**Conclusions:**

Our findings identify differences between AAV6 and AAV9 for myofiber-type preferences, which could be an underlying factor for mosaic transduction of skeletal muscle. Adjusting AAV serotype for specific muscle conditions can therefore improve transduction efficacy in clinical applications.

**Electronic supplementary material:**

The online version of this article (doi:10.1186/s13395-015-0064-4) contains supplementary material, which is available to authorized users.

## Background

Adeno-associated viruses (AAV) have been successfully engineered as gene-delivery vectors for efficient transduction of post-mitotic cells including skeletal muscles [1]. AAV-derived transgene expression is sustainable, consistent over time, and safe in preclinical applications [2]. Ongoing innovations in AAV vector engineering have further improved their suitability for application in clinical trials. Over the past decade, several AAV serotypes with variable tropism have been developed for superior transduction [3, 4], and most of these have been suggested for use in skeletal muscle transduction [5]. However, three AAV serotypes, AAV1, AAV6, and AAV9, are mostly used in pre-clinical studies [2]. Broad tropism, safety, and high transduction efficiency are among the main considerations for serotype utilization. However, this is not fully exploited and results are not always consistent between studies. For example, in dogs, AAV1 tropism is broader and not specific to skeletal muscles as compared to AAV9 [6], but AAV9 tropism in mice was suggested to be broader than AAV6 or AAV1 [2, 4]. Furthermore, in mice, AAV6 shows more resistance to proteasome-mediated degradation compared with AAV1 [7]. Elucidating tissue-specific tropism of AAV serotypes is therefore necessary to understand transduction efficiency and targeted application in the changing environment of a tissue like skeletal muscle.

Muscle tissue is composed of contractile fibers (also known as myofibers) that can be generally subdivided into four interchangeable types, marked by the expression of four myosin heavy chain (MyHC) isotypes: MyHC-2b, MyHC-2x, MyHC-2a, and MyHC-1 [8–10]. The distribution of the myofiber types changes dynamically to accommodate alterations in muscle function and metabolism [11]. Moreover, in muscular disorders, a switch in myofiber types is accompanied by muscle disuse and atrophic conditions [12, 13]. However, preference of AAVs for myofiber types and its contribution to overall transduction efficiency has not been thoroughly investigated. We studied myofiber-type preference in mouse tibialis anterior (TA) muscle following AAV6 or AAV9 intramuscular administration. We found that while AAV6 transduced smaller myofibers with a higher preference for MyHC-2x expressing myofibers, AAV9 transduction can be determined by MyHC expression. We therefore suggest that myofiber types of skeletal muscle can play a role in the selection of AAV serotypes for effective transduction and treatment.

## Methods

### AAV vectors

The AAV expression pTRCGW vector containing inverted terminal repeats (ITRs) of AAV2, EGFP (named here green fluorescence protein (GFP)) cDNA under control of the cytomegalovirus (CMV) promoter and polyadenylation signal of the simian virus 40, and the woodchuck post-transcriptional regulatory element (WPRE) was used for AAV production. AAV6 and AAV9 particles expressing the same vector were generated and purified as described before [14]. Briefly, for each AAV serotype, six 15-cm petri dishes containing 12.5 × 10^6^ HEK 293 T cells were transfected with branched polyethyleneimine (Sigma, St Louis, MO, USA). For AAV6, pTRCGW was co-transfected with the serotype-specific helper plasmid (ratio 3:1, total DNA 50 μg/plate). For AAV9, pTRCGW was co-transfected with the helper plasmid pAdΔF6 and the serotype-specific helper plasmid (ratio 2:2:1, total DNA 62.5 μg/plate). Helper plasmids for AAV6 were kindly provided by JA Kleinsschmidt [15] and for AAV9 by JM Wilson [16]. Transfected cells were grown in 20 ml Iscove’s modified Eagle’s medium with 10 % fetal calf serum (FCS), glutamate, and penicillin/streptomycin (Invitrogen, Carlsbad, CA, USA) for 2 days. Cells were lysed and virus particles were harvested and purified using a density gradient of Iodixanol as published before [17] with slight modifications. Briefly, cells containing virus particles were harvested into phosphate-buffered solution (PBS) and lysed by doing freeze-thaw in dry ice/ethonal for three times in the presence of DNAse (10 mg/ml). The lysate was incubated for an hour at 37 °C and centrifuged at 4000*g* for 30 min. The supernatant containing AAVs was then underlaid with a gradient of 15, 25, 40, and 60 % Iodixanol in water (Nycomed Pharma AS, Oslo, Norway) in Beckman Quick-Seal Polyallomer tube using a pasteur pipette. The tube was sealed and placed into a NVT90 rotor (Beckman Instruments) and centrifuged at 69,000 rpm for 70 min at 16 °C. Fractions of approximately 3 ml of the Iodixanol gradient (1 ml of 60 % layer and 2 ml of 40 % layer) were collected from the bottom of the tube. The AAV derived from the Iodixanol gradient was further diluted 10 times with PBS, pH 7.5, to reduce the viscosity of the Iodixanol, and was subsequently concentrated using Amicon Ultra-15 centrifugal filter units (Millipore, Amsterdam, The Netherlands). The removal of cellular impurities in AAV stocks were further confirmed using protein gel electrophoresis and electron microscopy as described before [17]. Virus titers were determined by quantitative PCR using a primer-set targeting the WPRE sequence of the expression cassette and indicated as genomic copies per unit volume (gc/ml) [14]. Genomic copies therefore represent only particles containing the expression cassette. All AAV stocks were kept at −80 °C prior to injections.

### Mouse strain and AAV particles injection

Male C57BL/6Jico mice of 7–8-week old were purchased from Jackson laboratories. After 1 week of acclimatization, AAV6 or AAV9 particles (2.13 × 10^10^ gc in 50 μl PBS; indicated in the text 2 × 10^10^ gc) were intramuscular injected into either left or right tibialis anterior muscles. Additionally, AAV9 (2.13 × 10^11^ gc; indicated in the text 2 × 10^11^ gc) and PBS control were injected into right or left TA muscles, respectively. Five mice were used per injection set. Injections were carried out under general anesthesia using 2 % isoflurane (Pharmachemie BV, Haarlem, The Netherlands). Mice were housed in ventilated cages with sterile bedding, water, rodent food, and air in DM-III containment level. Experiments were carried out in accordance with the Animal Research: Reporting of In Vivo Experiments (ARRIVE) guidelines [18]. An animal research protocol [#13113] was approved by the Institutional Animal Ethical Committee (DEC), Leiden University Medical Center, Leiden, the Netherlands.

### In vivo fluorescence imaging, muscle collection, in vitro imaging, and image quantification

After AAV injection, mice were imaged on a weekly basis for a period of 4 weeks using the Maestro™ in vivo fluorescence imaging system (Xenogen product from Caliper Life Sciences, Hopkinton, Massachusetts, USA) according to the manufacturer’s instructions. Mice were anesthetized with a continuous flow of 2 % isoflurane prior to imaging, and GFP fluorescence was acquired with an acquisition time ranging from 30 to 90 s depending upon the fluorescent signals from the AAV-injected TA muscles.

Four-week post-injection mice were sacrificed by cervical dislocation, and TA muscles were collected and immersed in liquid nitrogen cold isopentane for about 30–45 s and then stored at −80 °C for further processing. Transverse cryosections of 10-μm thick were made with a cryostat (Leica CM3050S) and pasted on superfrost plus glass slides (Menzel-Gläser; Thermo scientific) from four alternating parts across the muscle (Additional file 1: Figure S1). Additional tissue from the intervening areas was used for RNA isolation, ensuring representation of the whole muscle during data collection. Histological analyses of the muscle sections included hematoxylin and eosin staining [19] and immunofluorescence for myofiber typing. The untreated cryosections were directly incubated with a primary antibody to Laminin (1:1000; ab11575, Abcam). When indicated, a supernatant of hybridoma 6H1 (1:5, Developmental Studies Hybridoma Bank (DSHB), USA), detecting MyHC-2x was co-incubated. These antibodies were detected with secondary anti-rabbit-alexa-647 and anti-mouse-alexa-546 (Molecular Probes, Invitrogen). A mixture of monoclonal antibodies to MyHC-1, MyHC-2a, and MyHC-2b isotypes (hybridoma BA-D5, SC-71 and BF-F3, respectively (DSHB)), conjugated with alexa-350, alexa-594, and alexa-488, respectively was generated as describe before [8]. Antibody production from hybridomas and fluorophore conjugation was carried out as described earlier [10]. All antibody incubations were carried out in PBS containing 0.05 % tween (PBST) and 5 % dry milk, and washes were carried out with PBST. Slides were mounted with Aqua Polymount (Polyscience). Incubation with only anti-mouse conjugated secondary was carried out to exclude nonspecific binding to GFP positive myofibers. Imaging of GFP was carried out in untreated sections that were directly mounted with Polymount containing 4′,6-diamidino-2-phenylindole (DAPI). Imaging was carried out with either DM5500 or TCS-SP5 (confocal) microscopes (Leica) using LAS AF software versions: 2.3.6 (DM5500) and 2.5.1.6757 (TCS-SP5), respectively.

Image quantification was carried out with ImageJ, version 1.48 [20]. Per myofiber mean fluorescence intensity (MFI) and cross-sectional area (CSA) were documented after segmenting sections based on Laminin staining. Subsequently, MFI values were corrected for background and normalized for a fluorophore constant, which was determined from unbound fluorochrome per microscope and camera combination: (DM 5500: 430 nm = 0.735; 488 nm = 0.067; 596 nm = 0.172 and SP5: 488 nm = 0.003; 546 nm = 3.90).

### Quantitative RT-PCR

RNA isolation and RT-qPCR were performed as described previously [21]. Messenger RNA (mRNA) fold changes were calculated after normalizing to the average CT values of *Hprt* and *Gapdh* housekeeping genes and to the mean of PBS-injected TA muscles. Primers used in this study are listed in Additional file 1: Table S1.

### Statistical analyses

Fold changes and statistical significance of mRNA were determined with the Student’s *t* test in GraphPad Prism version 6.02. *P* values <0.05 were considered statistically significant. Statistical analyses of myofibers were carried out on natural log transformed values of the MFI (normalized to the fluorophore constant). A correlation between MFI of GFP and MyHC isotypes was assessed in a multivariate linear regression model with GFP MFI as a dependent variable and MyHC MFIs as independent variables. In a second multivariate model, CSA was added into the first model as an independent variable. Both models were stratified based on AAV serotypes. In addition, Pearson correlation was used to assess correlations between CSA, GFP, and the MFI of each MyHC isotype. Analyses were performed with IBM SPSS Statistics version 20.0.

## Results

### Enhanced transduction efficiency and inflammatory response by AAV6

**Fig. 1:**
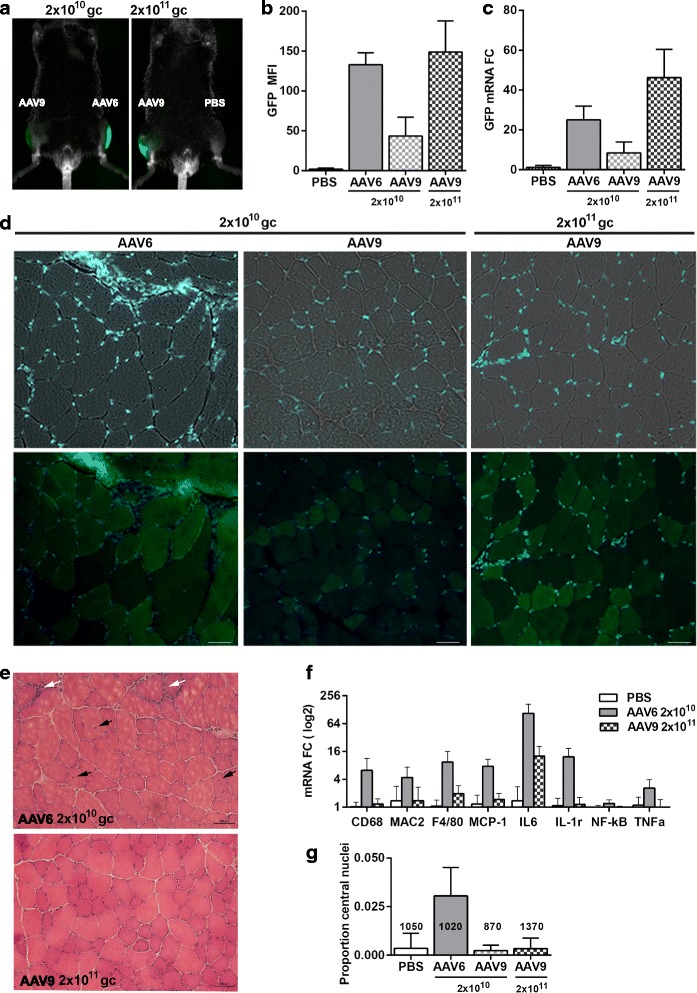
Intramuscular transduction efficiency and inflammatory responses by AAV6 and AAV9 serotypes. Identical dose of AAV6 or AAV9 particles (2 × 10^10^ gc) or AAV9 particles (2 × 10^11^ gc) and PBS control were injected into TA muscles. **a** Images of living mice show GFP in TA muscles 4 weeks post-injection. **b** GFP mean fluorescent intensity in TA muscles (*N* = 5). **c** GFP mRNA expression in the injected TA muscles (*N* = 5). CT values are normalized to *Hprt* and *Gapdh* housekeeping genes and to PBS-injected muscles. **d** Muscle histology of GFP expression in cross sections. *Upper row* shows bright field and DAPI staining of the nuclei. Images in the *lower row* were taken with a GFP filter and DAPI. Scale bar is 50 μm. **e** Muscle histology of HE staining. *White arrows* point to area with macrophage infiltration, and *black arrows* show myofibers with central nucleation. Scale bar is 200 μm. **f** Inflammatory gene expression in the AAV6- or AAV9-injected muscles (2 × 10^11^ gc) (*N* = 4). CT values are normalized to *Hprt* and *Gapdh* housekeeping genes and to PBS-injected muscles. All eight genes are significantly up-regulated in AAV6 muscles, but only IL6 is up-regulated in AAV9. **g** *Bar chart* shows the proportion of myofibers with central nuclei. The total number of fibers is indicated above each bar

To compare transduction efficacy identical doses (2 × 10^10^ gc) of AAV6 or AAV9 and a higher dose of AAV9 (2 × 10^11^gc), particles containing the same expression vector were injected into TA muscles. A contralateral PBS injection was used as a control. GFP expression was used to assess transduction efficiency in living animals on weekly basis. In all animals, the accumulation of GFP mean fluorescence intensity (MFI) was stabilized 3 weeks post-injection (Additional file 1: Figure S2), and the GFP signal was predominantly localized in TA muscles (Fig. 1a, Additional file 1: Figure S3). Mice were sacrificed at 4 weeks post-injection, and TA muscles were collected for the analyses. The GFP MFI measured from the AAV6 injected muscle was higher compared to that found in the AAV9 injected muscles with the same particle dose (Fig. 1a, b). Higher efficacy of AAV6 was also confirmed by GFP mRNA analysis (Fig. 1c). A 10-fold higher dose of AAV9 elevated the GFP MFI and mRNA as compared with AAV9 lower dose (Fig. 1b–d). In the AAV6 injected muscles, we observed remarkably high number of nucleated cells within the tissue, suggesting an activation of inflammatory response (Fig. 1e). Indeed, the expression of four macrophage markers (CD68, MAC2, F4/80, MCP-1) and four inflammatory markers (IL-6, IL1-R, NF-kB, and TNFa) was elevated in AAV6 as compared to PBS injected muscles (Fig. 1f). This observation is consistent with previous studies that suggest an induction of inflammatory responses by AAV6 [5, 22]. In contrast, in the high-dose AAV9-injected muscles, up-regulation of inflammatory genes was limited to IL6 (Fig. 1f), suggesting that inflammation in TA muscle is not activated by AAV9 administration.

Inflammation in muscles is associated with damaged muscles as characterized by leaky membranes and central nuclei [11]. In the AAV6-injected muscles, we found an increase in myofibers containing central nuclei (Fig. 1e and Additional file 1: Figure S4). About 3 % of myofibers in AAV6-injected TA muscles have central nuclei, but in the AAV9-injected muscles, the percentage of central nuclei was not different from the PBS injected muscles (Fig. 1g). Moreover, in the AAV6 but not in AAV9-injected muscles, we found regions with damaged myofibers and dispersed GFP fluorescence across myofibers (Additional file 1: Figure S5). This dispersed GFP fluorescence differed from a fiber-restricted localization of GFP in the AAV9-injected muscles (Fig. 1d).

### Preferential transduction of specific myofiber types by AAV6 and AAV9

**Fig. 2:**
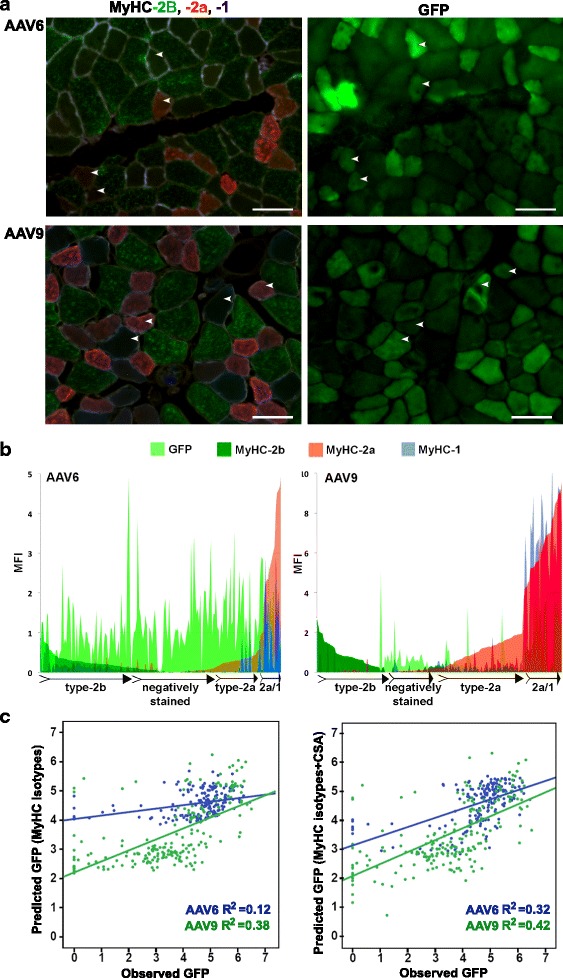
Analysis of GFP and myofiber-type correlation in AAV6- or AAV9-transduced muscles. **a** Images of representative sections after immunohistochemistry with four antibodies to MyHC isotypes (staining of each MyHC isotype separately is shown in Additional file 1: Figure S5) and GFP fluorescence in a consecutive section. Examples of matching myofibers are marked with *arrowheads*. Scale bar in 50 μm. **b** *Plots* show mean fluorescent intensity (MFI) distribution of MyHC isotypes and GFP within a myofiber (*N*_AAV6_ = 188 fibers; *N*_AAV9_ = 202 fibers); GFP in *light green*, MyHC-2b in *dark green*, MyHC-2a in *red*, and MyHC-1 in *blue*. Myofiber types that predominantly express MyHC-2b, MyHC-2a or MyHC-2a/1, and negatively stained myofibers are schematically indicated. **c** *Scatter plots* show the distribution of observed GFP MFI versus predicted GFP in AAV6 (in *green*) or AAV9 (in *blue*) conditions. Linear regression and fitness (*R* ^2^) are depicted in *green* or *blue* to AAV6 or AAV9, respectively. Predicted GFP is calculated using MyHC-2b, MyHC-2a, and MyHC-1 isotypes are variable (*left*) or MyHC-2b, MyHC-2a, and MyHC-1 isotypes and CSA (*right*)

Histological analysis revealed mosaic GFP expression in both AAV6- and AAV9-injected muscles. We investigated whether myofiber types could underpin differences in muscle transduction. We first assessed a simple correlation between GFP MFI and myofiber CSA. In both AAV6- and AAV9-transduced muscles, we found a negative correlation between GFP and myofiber CSA (Additional file 1: Table S2A). This negative correlation could partly be explained by higher concentration of GFP fluorescence in smaller myofibers. However, the correlation between CSA and GFP was much lower in AAV9-injected muscles compared with that of AAV6 (−0.39 and −0.55, respectively). Myofiber CSA was also correlated with the expression levels of MyHC isotypes (Additional file 1: Table S2A). We therefore further assessed whether AAV-mediated gene expression is affected by myofiber types; we correlated GFP expression per myofiber with the expression levels of myofiber types (Additional file 1: Figure S6). MFI of both GFP and each of MyHC fluorophores were measured per myofiber (Fig. 2a). Using a Pearson correlation, we found a negative correlation between MyHC-2b expression and GFP in both AAV6- and AAV9-injected muscles, whereas MyHC-2a expression positively correlated with GFP. The expression of MyHC-1 positively correlated with GFP in only AAV9-transduced muscles (Additional file 1: Table S2B). Plotting MFI of all four fluorophores per myofiber against GFP MFI of corresponding myofibers showed a negative preference of GFP expression in MyHC-2b expressing myofibers (Fig. 2b). Furthermore, we found a strong correlation between MyHC-2a and MyHC-1 expression in these muscles, but not between MyHC-2b and either MyHC-2a or MyHC-1 (Additional file 1: Table S2C). These analyses suggest hybrid myofibers co-expressing MyHC-1 and MyHC-2a; however, hybrid myofibers expressing MyHC-2b and either MyHC-2a or MyHC-1 were not identified in TA muscles.

Since we found hybrid myofibers co-expressing MyHC isotypes (Fig. 2b), we next assessed the contribution of each MyHC isotype to GFP expression using a multivariate model. In the AAV6-transduced muscles, a weak positive correlation was found between MyHC-2a expression and GFP (regression coefficient (*β*) = 0.25; *P* = 0.007; Table 1, Model 1). In the AAV9-transduced muscles, however, MyHC-2a expression negatively correlated with GFP whereas MyHC-1 positively correlated with GFP (*β* = −0.38, *P* < 0.001; *β* = 0.80, *P* < 0.001; respectively, Table 1, Model 1). The negative correlation between MyHC-2b and GFP remained unchanged in the multivariate model (Table 1, Model 1). As we found a correlation between GFP and CSA (Additional file 1: Table S2A), we then added CSA as an additional variable into the multivariate model. Surprisingly, in the AAV6-transduced muscles, only the CSA remained significant (Table 1, Model 2) whereas, in the AAV9-transduced muscles, a strong correlation between GFP expression and the aforementioned MyHC isotypes persisted (Table 1, Model 2). Scatter plots between the observed GFP and the GFP calculated from the multivariate models (predicted GFP) showed that in the AAV6- but not AAV9-transduced muscles, GFP MFI prediction was improved by threefold when CSA was added to the model (Table 1 and Fig. 2c). Together, these data suggest that AAV9-mediated transduction is determined by myofiber types whereas AAV6 transduction is determined primarily by myofiber size.

**Table 1:** MyHC protein expression correlates with AAV9- but not with AAV6-mediated transduction

	Model 1: Myofiber types				Model 2: Myofiber types and CSA			
	AAV6 (*N* = 185)		AAV9 (*N* = 202)		AAV6 (*N* = 185)		AAV9 (*N* = 202)	
	*β* for GFP (SE)	*P* value	*β* for GFP (SE)	*P* value	*β* for GFP (SE)	*P* value	*β* for GFP (SE)	*P* value
MyHC-2b	−0.12 (0.07)	0.070	−0.38 (0.06)	<0.001	−0.07 (0.06)	0.23	−0.27 (0.07)	<0.001
MyHC-2a	0.25 (0.09)	0.007	−0.24 (0.07)	<0.001	−0.15 (0.10)	0.12	−0.38 (0.08)	<0.001
MyHC-1	−0.38 (0.28)	0.170	0.80 (0.13)	<0.001	−0.03 (0.25)	0.91	0.84 (0.12)	<0.001

Linear regression analyses were performed including mean fluorescent intensities (MFI) for GFP as a dependent variable. In model 1, MFIs of myofibers expressing MyHC-2b, MyHC-2a, and MyHC-1 isotypes were included as independent variables. In model 2, cross-sectional area (CSA) of the myofibers was additionally included as an independent variable. Models were stratified for AAV6 and AAV9. Beta of the regression analysis and standard errors (SE) are provided

**Fig. 3:**
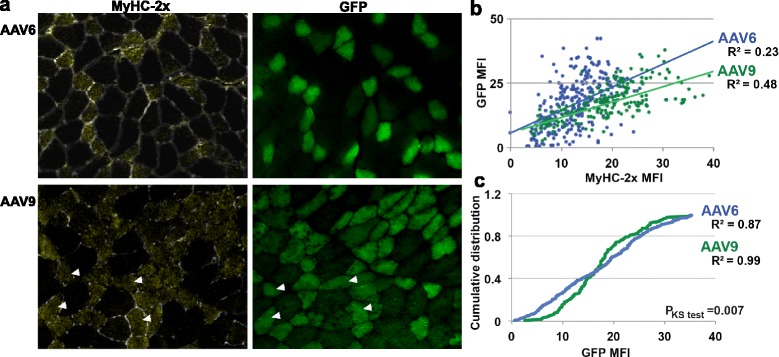
Analysis of MyHC-2x expression in GFP expressing myofibers. **a** Images of representative sections after immunohistochemistry with anti-MyHC-2x antibody and GFP fluorescence in the corresponding myofibers from a consecutive section. Examples of matching myofibers are marked with *arrowheads*. Scale bar is 50 μm. **b** *Scatter plot* shows a correlation between MFI of MyHC-2x and GFP in AA6 (in *blue*)- and AAV9 (in *green*)-transduced myofibers. Linear regression lines and fitness to the regression line (*R* ^2^) are indicated. **c**. Commutative correlation plot of GFP MFI in AA6 (in *blue*)- and AAV9 (in *green*)-transduced myofibers. Fitness to the regression line (*R* ^2^) and *P* value Kolmogorov–Smirnov (KS) test are indicated

The MFI distribution plots (Fig. 2b) suggest that GFP positive myofibers are enriched in the negatively stained population of myofibers. In this staining protocol, the negatively stained myofibers are regarded as MyHC-2x isotype expressing myofibers [8]. To assess whether GFP positive myofibers are indeed MyHC-2x expressing myofibers, we stained muscle sections with an antibody to MyHC-2x isotype (Fig. 3a). Primary antibody missing control was performed to exclude a possibility of preferential adherence of the conjugated secondary antibody alexa-546 with GFP positive myofibers (Additional file 1: Figure S7). A strong positive correlation was found between MyHC-2x expression and GFP in myofibers from AAV6- or AAV9-transduced muscles (Pearson correlation: *P* < 0.001) (Fig. 3b). Interestingly, a better fitness (>2-fold) to the linear regression line was found in the AAV9 muscles as compared with AAV6 (*R*^2^ = 0.48 or 0.23, respectively) (Fig. 3b). Commutative correlation plot of GFP MFI also confirmed better fitness to the regression line (*R*^2^) in AAV9-transduced myofiber compared with AAV6 fibers (Fig. 3c). These data suggest that both AAV6 and AAV9 show transduction preference for MyHC-2x expressing myofibers with a higher preference for AAV9.

## Discussion

In the last couple of decades, several AAV serotypes have been developed for effective gene delivery into various tissues including skeletal muscle with AAV vectors emerging as promising vehicles in clinical trials for monogenic musculoskeletal diseases including Duchenne and limb-girdle muscular dystrophy [23, 24]. Muscle tissue is composed of different but interchangeable myofibers, which determine muscle contraction and tissue stabilization. Here, we show that the transduction of myofiber types differs between AAV serotypes, using AAV6 and AAV9 as an example. The same expression vector was packaged in AAV6 and AAV9, and a standard protocol of virus particles generation and purification was used for both serotypes. This ensures that all observed differences are attributed to differences in AAV serotype. Consistent with previous studies, we found that AAV transduction of skeletal muscle results in a mosaic pattern [25–27]. Here, we found that the preferential transduction of myofiber types may explain the observed mosaic transduction by AAVs. A previous study has assessed correlation of AAV transduction with myofiber types using CMV-mediated *lac*Z as a reporter gene. Although this study found preferential transduction of the TA muscle over soleus, correlation of reporter gene expression with MyHC isotypes was unclear. The authors suggested that their procedure was limited by quantitative measurements [25]. We employed a quantitative histology procedure for myofiber typing by combining the antibodies to four MyHC isotypes. Moreover, in contrast to previous studies that used visual assessment for myofiber types [25, 26], our assessment of myofiber types is based on measurement of the MFI of each myofiber type. This quantification of MyHC isotypes expression per myofiber enables us to apply prediction models and determine the contribution of each myofiber type to transgene expression. We found significant differences between AAV6 and AAV9 for their transduction preferences to myofiber types. Myofibers expressing MyHC-2b isotype poorly express GFP that was derived by either AAV6 or AAV9. In our cassette, GFP was expressed under CMV promoter; thus, we cannot exclude the possibility of suboptimal activity of CMV promoter in MyHC-2b myofibers. However, previous studies showed that the CMV promoter does not discriminate between myofiber types [28, 29]. Additional studies with different promoters and reporters could clarify this potential concern. We suggest that not all myofiber types are readily permissive for AAV. In agreement with this hypothesis, a recent study demonstrated that AAV does not transduce satellite cells, the muscle stem cells within a muscle tissue [30]. In contrast to myofibers expressing MyHC-2b, myofibers expressing MyHC-2x isotype were preferentially transduced by both AAV6 and AAV9. Analysis of MyHC-2x was absent from a study that investigated the effect of AAV transduction between myofiber types [25]. Although, some receptors for AAV9 have been identified [31–33], but the molecular basis for AAV transduction and cell type specificity is still poorly understood. Elucidation of AAV serotype and myofiber specificity should be addressed in future studies, which should include screening of additional muscles expressing MyHC isotypes at different ratios and hybrid myofibers in different disease conditions. This will hopefully lead to develop more effective gene therapy strategies using AAV vectors.

Using the prediction model that includes myofiber types and CSA, we show that a correlation between GFP MFI and myofiber types was insignificant in the AAV6-transduced muscles but remained significant in the AAV9-transduced muscles. Importantly, addition of CSA to this model significantly improved the prediction of GFP MFI in AAV6- but not in AAV9-transduced muscles. Together, this suggests that AAV9-mediated transduction is predominantly determined by myofiber types whereas AAV6 transduction is determined primarily by myofiber size. Variations in transduction efficiency between AAV serotypes and different muscles are not fully understood. The TA muscle is more effectively transduced by AAV9 vector compared to the soleus muscle [26]. However, AAV6 is more effective for soleus transduction over AAV2 [25]. AAV1 transduction in dog models for muscular diseases seems to be more effective compared to mice [6]. Recently, AAV8 has also been suggested as an improved vehicle for transduction of muscle tissues [34]. Future studies should include testing of transduction efficiency of various serotypes in different muscles of multiple species, and this could result in an atlas of AAV serotypes and myofiber specificity for more effective utility of AAV-mediated gene therapy.

## Conclusions

In summary, we report that AAV-mediated myofiber transduction differs between AAV serotypes. Moreover, AAV serotypes show preferential transduction for myofiber types. We developed a quantitative histology procedure for myofiber typing, which allows us to assess AAV-mediated transduction using statistical prediction models. Using these models, we show that in skeletal muscle, fast-twitch myofiber expressing MyHC-2b isotype is poorly transduced, whereas those expressing MyHC-2x isotype are preferentially transduced by both AAV6 and AAV9. We further show that in wild-type mice, transduction of myofibers by AAV9 serotype can predominantly be explained by myofiber type, whereas transduction by AAV6 serotype can be explained by myofiber size, but not by myofiber type. Future studies should expand these results in muscular disease models, and such studies may open a new discussion to the choice of AAV serotype and cell type specificity for AAV-mediated transduction in clinical applications. We suggest that understanding of AAV tropism at myofiber type levels will improve transduction efficacy and specificity for clinically interesting muscles.

## Additional file

Additional file 1:
**A pdf file containing two supplementary tables (S1–2) and five supplementary figures (S1–5) are included as an Additional file** **1** **.** **Table S1** contains primer sequences used in this study. **Table S2** contains myofiber-based univariate correlations. **Figure S1** shows schematic summary of methodology. **Figure S2** shows GFP fluorescence in AAV-injected TA muscles. **Figure S3** shows GFP fluorescence in all 10 AAV- or PBS-injected mice at 4 week post-injection. **Figure S4** indicates myofiber damage in AAV6-transduced TA muscles. **Figure S5** shows immunohistochemistry with antibodies to MyHC type-2b, MyHC type-2a, and MyHC type-1 and to laminin in AAV6- and AAV9-injected animals.

## Abbreviations

AAV: adeno-associated virus
ARRIVE: Animal Research: Reporting of In Vivo Experiments
CMV: cytomegalovirus
CSA: cross-sectional area
CT: cycle of threshold
DAPI: 4′,6-diamidino-2-phenylindole
DEC: Dier Experimenten Commissie (Animal Ethical Committee)
DSHB: developmental studies hybridoma bank
EGFP: enhanced green fluorescence protein
FCS: fetal calf serum
Gapdh: glyceraldehyde-3 phosphate dehydrogenase
GFP: green fluorescence protein
HEK cells: human embryonic kidney cells
Hprt: hypoxanthine phosphoribosyltransferase
ITRs: inverted terminal repeats
LAS AF: leica application suite advanced fluorescence
MD: muscular dystrophies
MyHC: myosin heavy chain
MFI: mean fluorescence intensity
PBS: phosphate-buffered solution
PBT: PBS containing 0.05 % tween
RT-qPCR: reverse transcriptase quantitative polymerase chain reaction
SE: standard error
TA: tibialis anterior
WPRE: woodchuck post-transcriptional regulatory element
