# Supplementary material for: Differential myofiber-type transduction preference of adeno-associated virus serotypes 6 and 9
Source: Skelet Muscle. 2015 Nov 10;5:37. doi: 10.1186/s13395-015-0064-4 (PMC4641337; doi:10.1186/s13395-015-0064-4)
Supplement: Additional file 1: — A pdf file containing two supplementary tables (S1–2) and five supplementary figures (S1–5) are included as an Additional file 1 . Table S1 contains primer sequences used in this study. Table S2 contains myofiber-based univariate correlations. Figure S1 shows schematic summary of methodology. Figure S2 shows GFP fluorescence in AAV-injected TA muscles. Figure S3 shows GFP fluorescence in all 10 AAV- or PBS-injected mice at 4 week post-injection. Figure S4 indicates myofiber damage in AAV6-transduced TA muscles. Figure S5 shows immunohistochemistry with antibodies to MyHC type-2b, MyHC type-2a, and MyHC type-1 and to laminin in AAV6- and AAV9-injected animals. [file 13395_2015_64_MOESM1_ESM.pdf]

SUPPLEMENTARY MATERIALS

Supplementary Table S1: Primer sequences used in this study.

Gene name	qPCR primer sequences (5' to 3')	
	Forward	Reverse
Myh1	ACCTGGCCAAATTCCGCAAG	TGGTCACTTTCCTGCACTTG
Myh2	TGAACTGGAGGGTGAGGTAG	TCTGGTAAGTAAGCTCCTTC
Myh4	GAGTGAAGGAACTCACCTAC	TTTCACTTTAGTCTGTAGTTTG
Myh7	GGCAACTGAGTCACCTAAGCT	AGGGCTTGCTCATCCTCAAT
Tnnt1	GCTGGGAAGGGTCGAGTTG	GGGCACTTTATTTTGAGTTAC
Hprt	CCCTGGTTAAGCAGTACAGCC	CGAGAGGTCCTTTTCACCAGC
Gapdh	CAACGAATTTGGCTACAGCA	AGGGGTCTACATGGCAACTG
Cd68	CCAATTCAGGGTGGAAGAAA	GAGAGAGACAGGTGGGGATG
Mac2	GATCACAATCATGGGCACAG	AAGGGGAAGGCTGACTGTCT
F4/80	CTTTGGCTATGGGCTTCCAGTC	GCAAGGAGGACAGAGTTTATCGTG
MCP-1	CACTCACCTGCTGCTACTCA	GCTTGGTGACAAAAACTACAGC
IL6	ACCACGGCCTTCCCTACTTC	CTCATTTCCACGATTTCCCAG
IL-1r	TCAGAATCTGGGATACTAACC	CTAGTTTGATATTTGGTCCTTG
NF-kB	CAGCTTTACAGAGTCTCTTAC	CTTTGGATTCGCTTTGCCTTC
TNFα	GATCGGTCCCCAAAGGGATG	CACTTGGTGGTTTGCTACGAC

Supplementary Table S2: Myofiber-based univariate correlations. Correlations were calculated with a simple regression model as indicated in each table. AAV6 N_{fibers}=188 (GFP, MyHC type-2b, type-2a, type-1) or N_{fibers}=208 (MyHC type-2x); AAV9 N_{fibers}=202 (GFP, MyHC type-2b, type-2a, type-1) or N_{fibers}=206 (MyHC type-2x).

Supplementary Table S2a

**CSA**	**AAV6**		**AAV9**	
	R	p-value	R	p-value
**GFP**	-0.55	<0.001	-0.39	<0.001
**MyHC-2b**	0.46	<0.001	0.58	<0.001
**MyHC-2a**	-0.67	<0.001	-0.64	<0.001
**MyHC-1**	-0.23	<0.001	-0.42	<0.001
**MyHC-2x**	-0.47	<0.001	-0.65	<0.001

Table shows Pearson correlation (R) and significance (p-value, 2-tailed) between fiber cross-sectional area and MyHC isotypes expression in AAV6 or AAV9 injected TA muscles. Positive or negative correlations are depicted in black or red, respectively.

Supplementary Table S2b

**GFP**	**AAV6**		**AAV9**	
	R	p-value	R	p-value
**MyHC-2b**	-0.29	<0.001	-0.50	<0.001
**MyHC-2a**	0.30	<0.001	0.18	<0.01
**MyHC-1**	0.06	N.S.	0.48	<0.001

Table shows Pearson correlation (R) and significance (p-value, 2-tailed) between GFP MFI and MyHC isotypes expression in AAV6 or AAV9 injected TA muscles. Positive or negative correlations are depicted in black or red, respectively. N.S. is non-significant.

Supplementary Table S2c

	**AAV6**				**AAV9**			
	**MyHC-2a**		**MyHC-1**		**MyHC-2a**		**MyHC-1**	
	R	p-value	R	p-value	R	p-value	R	p-value
**MyHC-2b**	-0.55	<0.001	-0.16	0.03	-0.38	<0.001	-0.43	<0.001
**MyHC-2a**			0.54	<0.001			0.63	<0.001

Table shows Pearson correlation (R) and significance (p-value, 2-tailed) between every two MyHC expression per myofiber in AAV6 or AAV9 injected TA muscles. Positive or negative correlations are depicted in black or red, respectively.

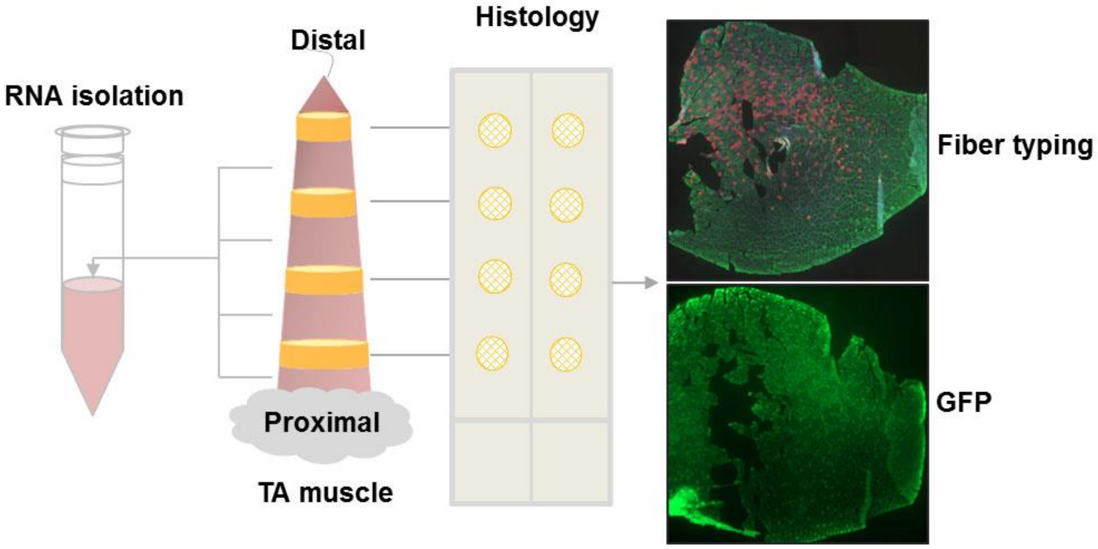

Supplementary Figure S1: Schematic summary of methodology. The injected TA muscles were harvested 4 weeks post injection. Tissue collections for histology or RNA extraction alternates four times along the muscles from the distal side, ensuring representation of the whole muscle in each procedure. Examples of images for histological analyses from GFP or immunohistochemistry of myofiber typing are shown.

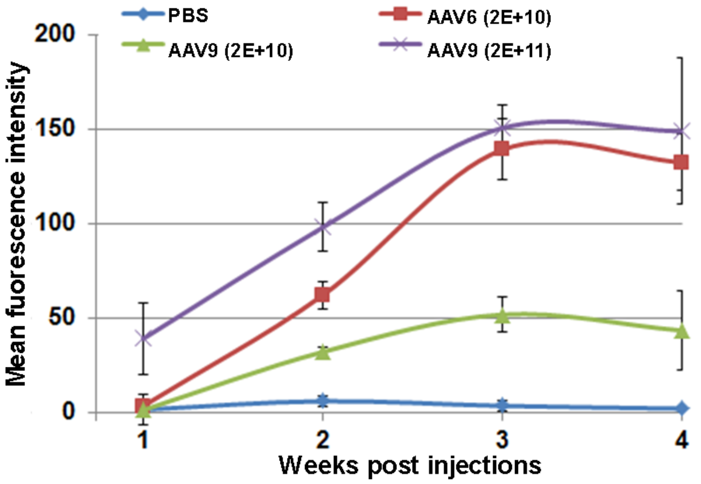

Supplementary Figure S2: GFP fluorescence in AAVs injected TA muscles. Plot shows GFP accumulation in TA muscles over 4 weeks. All imaging were carried out with identical settings. MFI (mean fluorescence intensity) within TA muscle was measured with ImageJ. Averages and SD are from 5 animals.

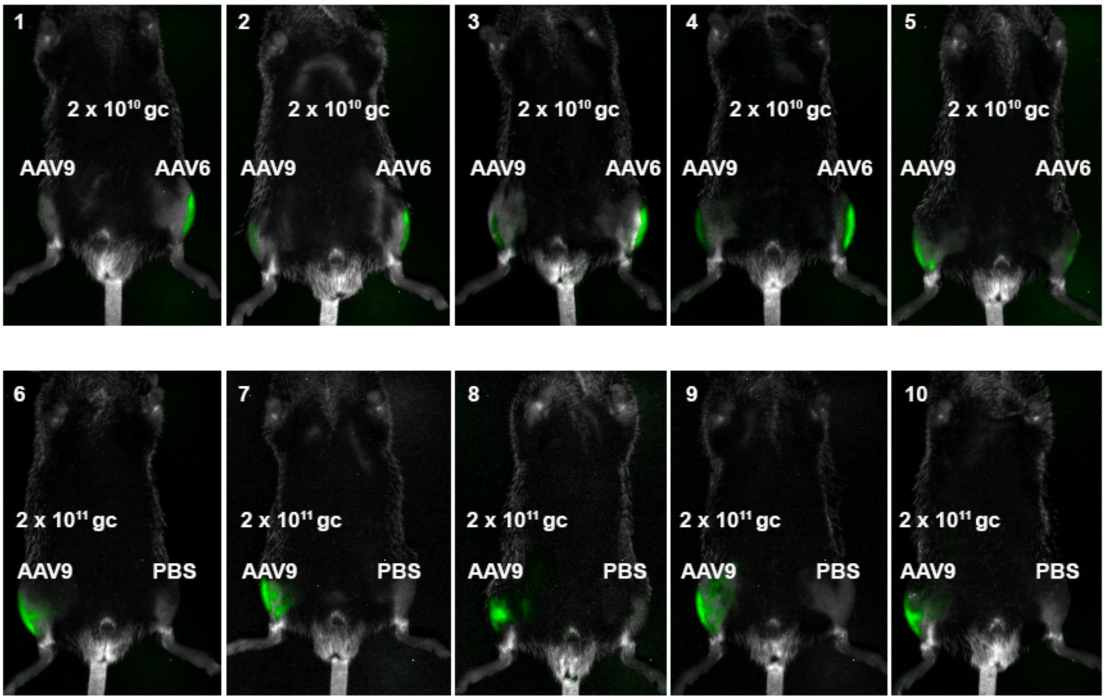

Supplementary Figure S3: GFP fluorescence in all 10 AAV- or PBS-injected mice 4 weeks post injection. Images show mice and GFP fluorescence 4 weeks after AAV injection. Upper row (mice # 1-5) shows GFP expression in TA injected with a similar dosage of AAV6 or AAV9 (2 x10^{10} gc). In mouse # 5, the serotype injection was switched. Lower row (mice # 6-10) shows GFP expression in TA injected with AAV9 at a 10 fold higher dose (2 x10^{10} gc) or PBS injected muscles.

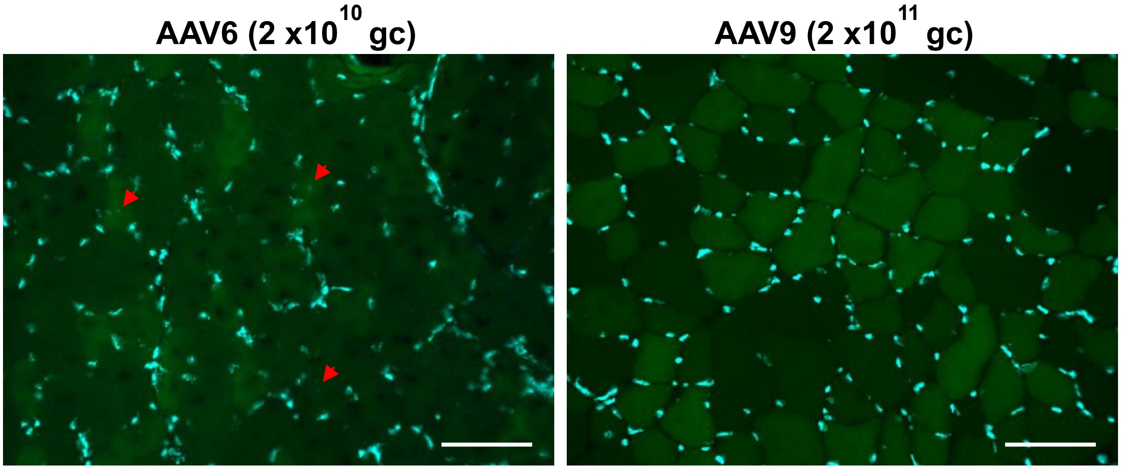

Supplementary Figure S4: Myofibers with central nucleation. AAV6 injected myofibers show central nucleation as compared with AAV9 injected myofibers. Sections were only stained with DAPI and images were taken with 20X objective. Scale bar is 50 µm. The myofibers with central nucleation are indicated with red arrows.

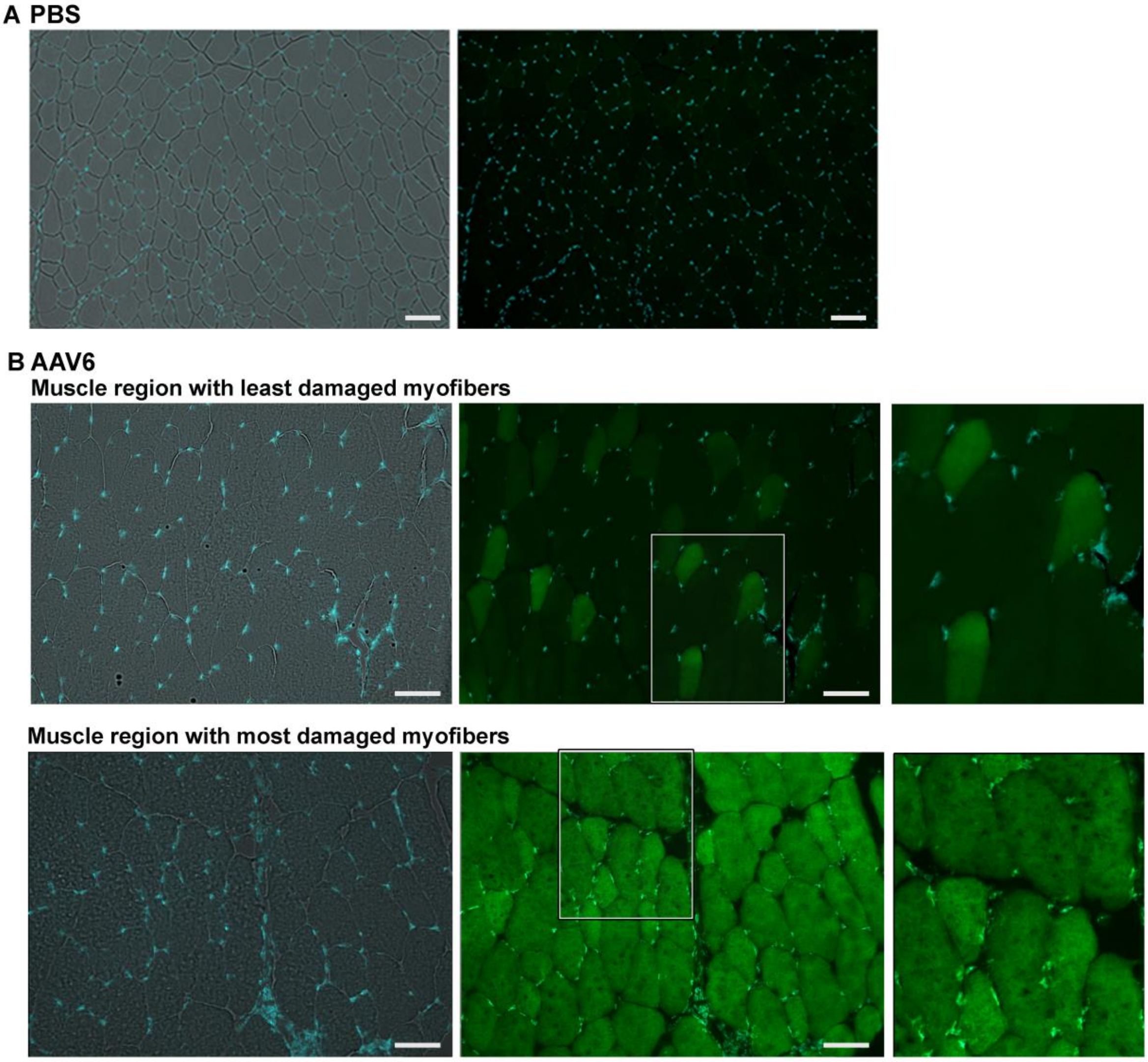

Supplementary Figure S5: Myofiber damage in AAV6-transduced *TA* muscles. A representative cross section of TA muscles injected with PBS. Left image shows bright field + DAPI image. Right image shows GFP+DAPI. B. Cross sections from AAV injected TA. The upper panel shows a representative area with a myofiber-confined GFP. The lower panel shows a representative area with GFP fluorescence that is evenly distributed across fibers. Higher magnification (left) reveals damaged fibers with black holes. Inflammation is also pronounced in this part of the tissue. Left images show bright-field overlay with DAPI. Right images show a zoom into the boxed were made with a 63X objective. Scale bar is 50 µm.

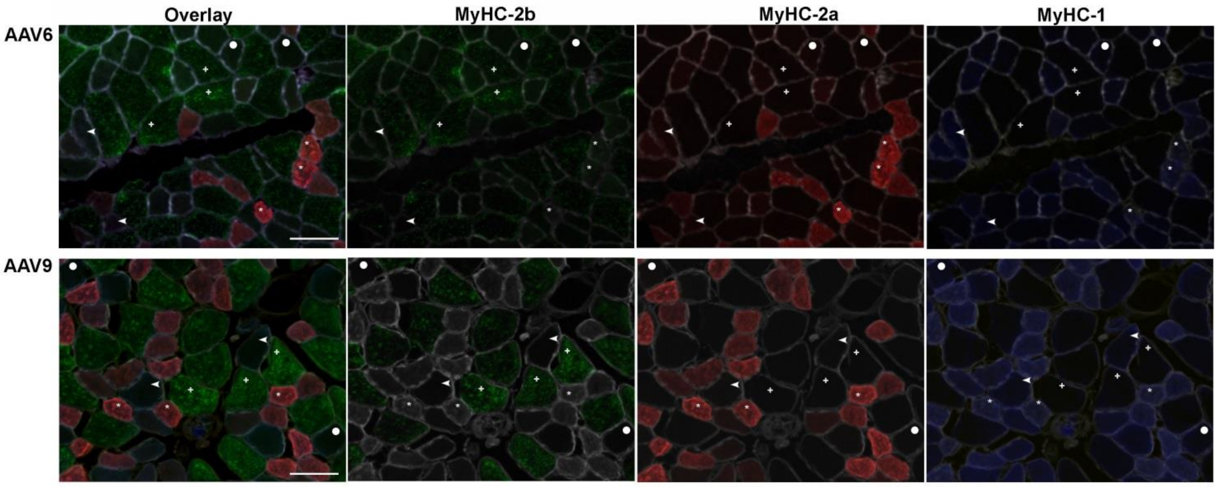

Supplementary Figure S6: Immunohistochemistry with antibodies to MyHC type-2b, -2a, -1 and to laminin in AAV6- and AAV9-injected animals. Images show representative cross-sections of immunofluorescence staining for MyHC isotypes in AAV6 or AAV9-injected TA muscles. Left panel shows overlay images, and right panel shows separate channels for each MyHC isotype (MyHC-2a in red, MyHC-1 in blue and MyHC-2b in green). Fibers are counterstained with Laminin. Scale bar is 50 µm. Corresponding fibers are marked with arrow heads, plus signs, asterisks, and circles indicating myofibers with predominant expression of type-1, type-2b; type-2a and co-expression of type-1 and type-2a, respectively, in a stained section with scale bar 50µm.

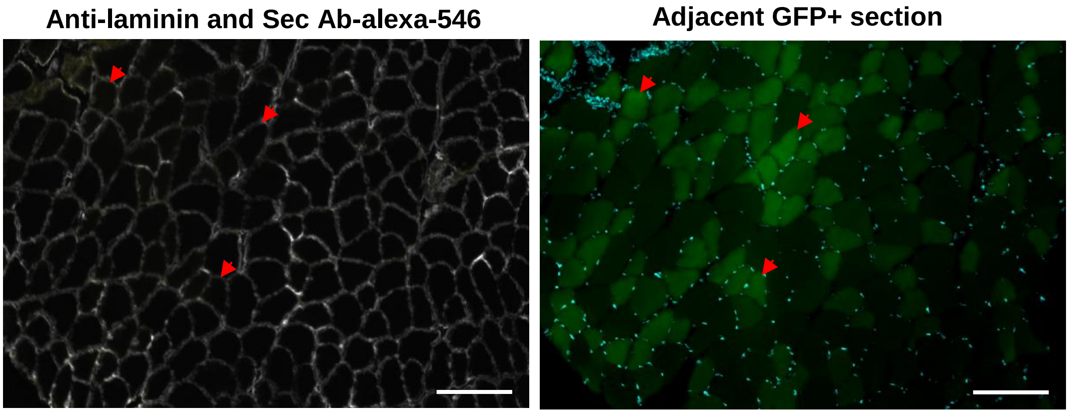

Supplementary Figure S7: Primary antibody (6H1 against type-2x) missing control. The section was first stained for laminin to visualize myofiber boundaries and then incubated with secondary antibody conjugated with alexa-546 fluorophore without prior incubating with 6H1 primary antibody against type-2x myofibers. No fluorescence signals of alexa-546 were observed in the corresponding GFP positive myofibers of adjacent section. The adjacent section is stained with only DAPI and images are taken with a 10X objective. Scale bar is 50 µm. The corresponding myofibers are indicated with red arrows.
